# Neurometabolic Response to Apneic Stimuli Tracks Global Grey Matter Volume Deficits in Patients With Obstructive Sleep Apnea

**DOI:** 10.1111/jsr.70250

**Published:** 2025-11-19

**Authors:** Jeffrey B. Dennison, Pei‐Hsin Wu, Michael C. Langham, Richard J. Schwab, John A. Detre, Felix W. Wehrli

**Affiliations:** ^1^ Department of Radiology, Perelman School of Medicine University of Pennsylvania Philadelphia Pennsylvania USA; ^2^ Department of Electrical Engineering National Sun Yat‐sen University Kaohsiung Taiwan; ^3^ Division of Sleep Medicine, Department of Medicine University of Pennsylvania Philadelphia Pennsylvania USA; ^4^ Department of Neurology University of Pennsylvania Philadelphia Pennsylvania USA

**Keywords:** cerebral oxygen metabolism, grey matter volume fraction, sleep apnea

## Abstract

Obstructive sleep apnea (OSA) has been linked to cortical atrophy and increased risk of neurodegeneration, a process potentially mediated by neurometabolic dysregulation. Recent work using high temporal‐resolution metabolic MRI found greater changes in the cerebral metabolic rate of oxygen consumption (CMRO_2_) during cued breath‐hold stimuli simulating spontaneous apneas in OSA patients than in healthy people. Here, we conducted a secondary analysis on this work to assess whether the observed neurometabolic responses were associated with grey matter volume fraction. We examined previously collected data from OSA patients matched to non‐OSA participants by age and sex to evaluate associations between MRI‐based measures of neurometabolism and grey matter volume. Grey matter volume fraction (GMVF) was computed by segmenting T1‐weighted structural images and regressing out age effects in OSA patients relative to the age‐matched non‐OSA participants. We then examined the relationship between GMVF, apnea‐hypopnea index (AHI), and neurometabolic responses of cerebral blood flow (CBF), venous oxygen saturation (SvO_2_), and CMRO_2_ in response to breath‐hold stimuli. OSA severity, expressed in terms of AHI, was negatively associated with GMVF in both OSA and non‐OSA reference subjects after controlling for age. Incorporating the quantitative measures of brain oxygen metabolism showed breath‐hold responses in CBF and SvO_2_ to be positively associated with GMVF. However, changes in CMRO_2_ were not significantly associated with age‐normalized GMVF. The findings suggest that noninvasive global measures of brain oxygen metabolism may serve as quantitative markers of neural health. Further research is warranted to explore these measures and neurodegenerative risk in OSA.

## 1 Introduction

Obstructive sleep apnea (OSA) is a highly prevalent sleep disorder (Simpson et al. 2013; Benjafield et al. 2019) characterized by recurrent interruptions of airflow (apneas and hypopneas (Eckert 2018)) during sleep causing transient reductions in arterial oxygen saturation of at least 3% (Lavie 2015; Berry, et al. 2014). The ensuing sleep disturbances adversely affect patients' quality of life (Bjornsdottir et al. 2015). Further, OSA has been found to be associated with neurocognitive deficits (Bucks et al. 2013) as well as reduced grey matter volume (Joo et al. 2010; Shi et al. 2017; Kim et al. 2022), though regional variations and conflicting findings have also been noted (Baril et al. 2021). At the worst, OSA is associated with increased risk of neurodegenerative disorders including Alzheimer's (Andrade et al. 2018) and Parkinson's (Sun et al. 2020) disease, and it has been suggested this increase may be mediated by dysregulation of neurometabolism (Lavie 2015) However, establishing a direct link between brain structural manifestations and neurometabolic dysregulation in OSA poses significant challenges largely due to the technical difficulties of quantitatively assessing neurometabolism in situ and during sleep.

In recent work Rodgers et al. (2016), and subsequently in a larger study, Wu et al. (2022) used a rapid quantitative MRI technique to quantify venous oxygen saturation (SvO_2_) and cerebral blood flow (CBF) at a temporal resolution on the order of two seconds, from which the cerebral metabolic rate of oxygen (CMRO_2_) can be computed via Fick's principle. As opposed to observing spontaneous apneas during sleep, OSA patients performed cued breath‐hold stimuli to simulate apneic events during sleep. Such simulated apneas engender a hypocapnic‐hypoxic response consisting of a transient increase in CBF and an increase in SvO_2_. These neurometabolic changes may reflect a failure in the autoregulation of disorders such as ischemia and degenerative disease (Ishii et al. 1996; An et al. 2009). The breath‐hold stimulus is not isometabolic as previously suggested (Rodgers et al. 2013) but rather, the observed increase in CMRO_2_ during a cued breath‐hold has been found to be enhanced in patients with OSA relative to non‐OSA and healthy young reference subjects (Wu et al. 2022).

In this retrospective study we re‐examined the data from Wu et al. (2022) to evaluate the hypothesis that the neurometabolic stimulus response to induced apneas (repeated 24‐s breath‐hold challenges) is associated with brain volume changes based on the increases in CMRO_2_ in OSA patients. Wu et al. had collected 3D high‐resolution T1‐weighted images of the brain to determine brain mass (CMRO_2_ is expressed by μmol O_2_/min/100 g brain mass) making this assessment possible.

We evaluated the associations between (1) OSA severity as measured by the apnea‐hypopnea index (AHI), (2) the neurometabolic apneic response, and (3) grey matter volume fraction (GMVF). We first evaluated the relationship between AHI and GMVF, controlling for age. We then evaluated the relationship between grey matter and neurometabolic parameters, including SvO_2_, CBF, and CMRO_2_ at baseline and during successive cued breath‐hold challenges. We conjectured that changes to neurometabolic parameters in response to breath holds, mimicking the hypoxic conditions of naturally occurring spontaneous apneas, are predictive of the grey matter loss experienced by the OSA participants previously evaluated.

## 2 Methods

For each participant in this secondary analysis, a GMVF was determined. Associations were then examined between neurometabolic parameters at baseline and in response to breath‐holds, reported previously. Strengthening the reporting of observational studies in epidemiology (STROBE) guidelines was followed by the authors.

### 2.1 Participants

The previously reported study (Wu et al. 2022) included OSA patients (*N* = 30) and non‐sleep apnea (NSA) participants (*N* = 22) matched for age, height, weight, and body mass index (BMI). The experimental protocol for the original study was approved by the University of Pennsylvania's Institutional Review Board and informed consent was obtained from all study subjects. Participants ranged in age from 30 to 70 years of age (OSA mean age = 49.5 sd = 10.6; NSA mean age = 49.0 sd = 10.6) with an AHI of 0.2–124.5 (OSA mean = 44.8 sd = 25.0; NSA mean = 2.6 sd = 2.5). Both OSA and NSA subjects were drawn from a pool of patients with abnormal sleep patterns, examined at the University of Pennsylvania's Sleep Center between February 2016 and November 2019, and grouped on the basis of the apnea hypopnea index (AHI) from polysomnography. OSA was classified as AHI > 15 events/h and NSA as AHI < 10 events/h. Other symptoms of OSA (e.g., daytime sleepiness, snoring) were not systematically collected. Participants were screened for standard MRI exclusion criteria (claustrophobia, pregnancy, metal implants, etc.) and significant neurological conditions likely to affect cerebral metabolism and blood flow, including congestive heart failure, chronic obstructive pulmonary disease, stroke, and head trauma. Cigarette smokers and users of other nicotine products were also excluded.

Patient demographics and diagnosis used to compare OSA and NSA are reported in Table 1 below and include the AHI and SaO_2_ nadir obtained during diagnostic work‐up at the authors' institution Sleep Center. An additional eight subjects were removed from analyses due to excessive motion during the neurometabolic portion of the scan or difficulty performing the breath‐hold apnea, leaving 44 participants for the final analyses. Additional details on enrollment and exclusions can be found in Wu et al. (2022). As this project represents a secondary analysis, no additional power analyses were conducted on our ability to detect a relationship between OSA, neurometabolism, and grey matter volume.

**TABLE 1:** Demographics of study participants and diagnostic information.

Variable	OSA	NSA	*p* value
% Female	40%	23%	*p* = 0.34
Age	49.5 ± 10.6	49.0 ± 10.6	*p* = 0.84
Height (inches)	68.5 ± 3.3	68.5 ± 3.7	*p* = 0.94
Weight (lbs)	207.8 ± 37.7	202.7 ± 36.0	*p* = 0.64
BMI (kg/m^2^)	30.1 ± 4.3	31.2 ± 3.3	*p* = 0.85
AHI (events/h)	39.5 ± 20.4	2.6 ± 2.4	*p* < 0.001
SaO_2_ Nadir (%HbO_2_)	74 ± 11	87 ± 4	*p* < 0.001

*Note:* The right most column shows the associated *p* values from a χ^2^ test of independence for % female and independent sample t‐tests for age, height, weight, BMI, AHI, and SAO_2_ Nadir.

### 2.2 Study Protocol

**FIGURE 1:**
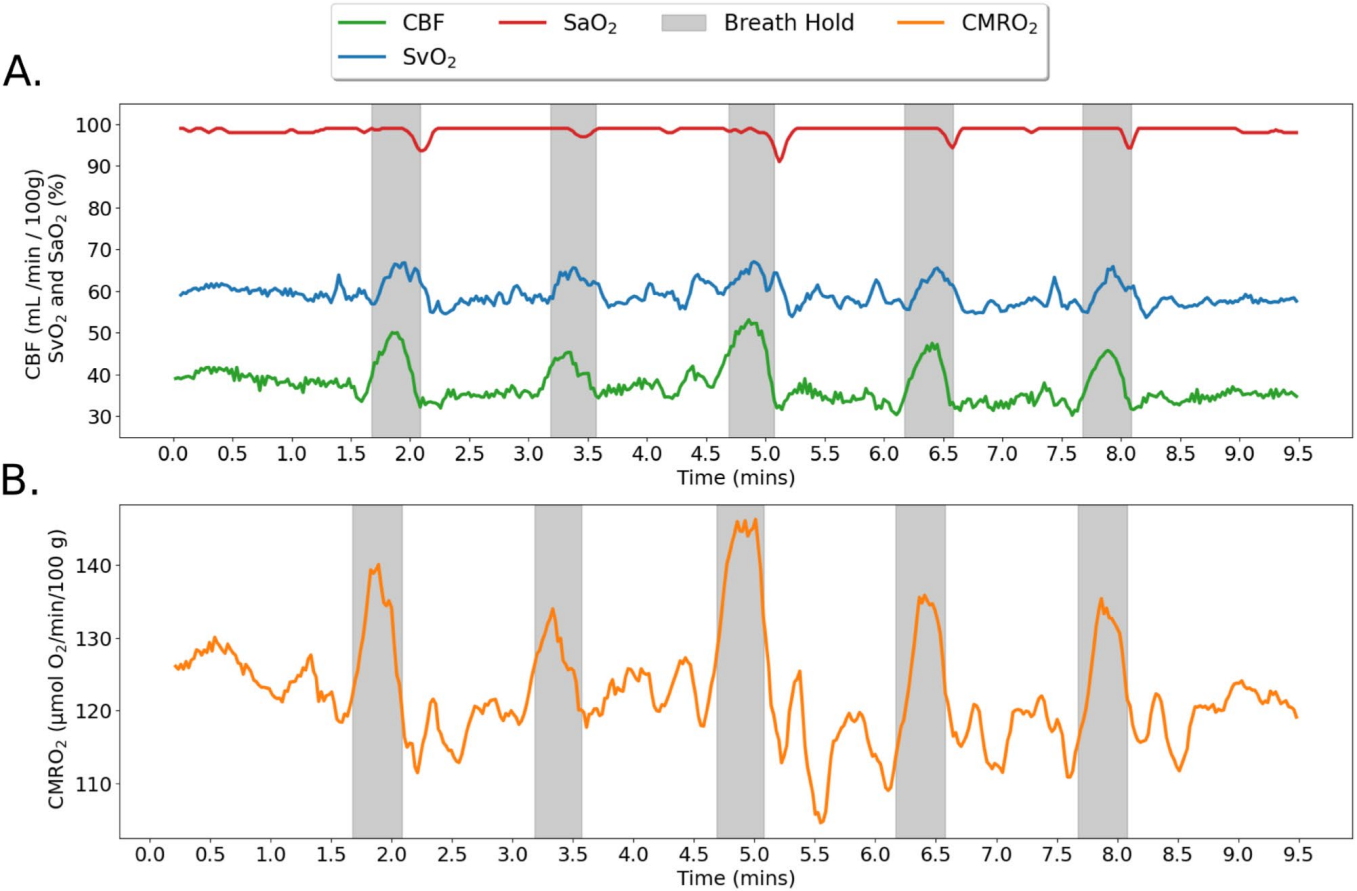
Vascular‐metabolic parameters plotted were created from the raw data of a single participant (male, 69 years of age) in Wu et al. (2022). Periods of suspended respiration (breath‐hold) are shown in grey. Changes in SaO_2_ (red), SvO_2_ (blue), and CBF (green) are displayed in panel A, those in CMRO_2_ (orange), computed via Equation (1), are shown in panel B. For display purposes, these parameters have been temporally smoothed with a moving average strategy.

In the work by Wu et al. (2022) previously conducted in the authors' lab, participants had been scanned by 3 Tesla magnetic resonance imaging of the brain, for 15 min at 2.3 s temporal resolution, yielding time‐resolved whole‐brain cerebral blood flow and venous oxygen saturation. During scanning participants followed a breath‐hold paradigm so that temporal changes to cerebral blood flow and oxygenation could be measured. To mimic the hypercapnic‐hypoxic conditions of an apnea experienced in OSA, after an initial 100‐s baseline period of normal breathing, participants underwent a series of five 24‐s volitional apnea challenges (breath‐holds), each followed by 66 s of normal breathing. Visual instructions were presented by projecting commands onto a screen with pre‐recorded verbal instructions given via MRI‐compatible headphones. The breath‐hold challenge evokes a hypercapnic‐hypoxic response analogous to that in spontaneous apneas during sleep, resulting in an increase in CBF and SvO_2_ along with a small decrease in SaO_2_ (due to transient hypoxemia) (see Figure 1). The three measured variables then yield global CMRO_2_ in response to the stimulus via Equation (1) below in absolute physiologic units.

### 2.3 Data Analysis

#### 2.3.1 Structural Analysis

Structural T_1_‐weighted MPRAGE images were corrected for intensity non‐uniformity (INU) with Advanced Normalization Tools 2.5.3 (Avants et al. 2008) (RRID:SCR_004757) N4BiasFieldCorrection (Tustison et al. 2010) and used as the reference image throughout the workflow. The reference was skull‐stripped with a Nipype implementation of the antsBrainExtraction.sh workflow (from ANTs), using OASIS30ANTs as the target template. Brain tissue segmentation of cerebrospinal fluid (CSF), white matter (WM), and grey matter (GM) was performed on the brain‐extracted T1w using fast (FSL 6.0.7.8), RRID:SCR_002823, (Zhang et al. 2001).

Grey matter volumes were then obtained by thresholding probabilistic grey matter maps at 50% (Wang et al. 2018). In this manner a voxel that is more likely grey matter than not is assigned to grey matter. The volume of the grey matter mask was then normalized by intracranial volume by dividing by the volume of the total brain mask as in previous methods (Nakamura and Fisher 2009).

#### 2.3.2 Cerebral Rate of Oxygen Metabolism via Fick's Principle

CMRO_2_ was quantified using the conservation of mass or Fick's Principle (Kety and Schmidt 1948):

(1) $${\text{CMRO}}_{2}=\mathrm{Ca}\times \mathrm{CBF}\times {\mathrm{SaO}}_{2}-{\mathrm{SvO}}_{2}$$

Here, C_a_ is the oxygen carrying capacity of red blood cells (1.39 mL O_2_/g [Hb]) (Cao et al. 2018). Subject‐specific haemoglobin levels (Hb) were made available from measurements via a fingerstick blood sample (Hemocue Hb 201+, Angelholm, Sweden) and SaO_2_ was monitored via digital pulse oximetry (Medrad Veris 8600, Bayer Medical Care Inc., Indianola, PA, USA). The final parameters, CBF and SvO_2_ (venous oxygen saturation) had been obtained via the high temporal resolution OxFlow imaging method (Caporale et al. 2023) in the superior sagittal sinus, the major draining vein of the brain.

#### 2.3.3 Neurometabolic Time‐Series

For each participant a time series (see Figure 1 for an example) of cerebral blood flow (CBF) and venous oxygenation (SvO_2_) was available from the data in Wu et al. (2022), to which the reader is referred for a detailed account of the data processing. In brief, data for CBF and SvO_2_ were obtained from velocity and field maps using the average values within the SSS. Both velocity and field maps had been reconstructed offline using a set of custom scripts written in Matlab. Field maps had been calculated by taking the phase difference between two GREs with different TEs, while the phase difference between two GREs with the same TE but equal and opposite velocity‐encoding results in a velocity map with appropriate scaling.

To calculate whole‐brain CBF, the flow rate measured in the SSS was upscaled using the ratio between blood flow rate in the SSS and the combined blood flow rate from the internal carotid and vertebral arteries, with the latter having been measured at the beginning of the time series (Caporale et al. 2023). Finally, the result was normalized by brain mass, determined by multiplying 1.05 g/mL by the brain volume computed from the images acquired with MP‐RAGE.

From the field maps, the average difference in the local field inside and outside the SSS can be obtained, with the difference being linearly proportional to SvO_2_, that is, the induced magnetic field of whole blood is related to the blood oxygenation level. Calculations of SvO_2_ also incorporated individual haemoglobin levels assessed through finger prick measures (see Jain et al. 2010 for details on the calculation).

### 2.4 Regression Analysis

First, we aimed to replicate the previously shown negative relationships between age and normalized grey matter volume (Taki et al. 2011) and to evaluate a possible association between OSA severity and grey matter volume, as previously observed by Joo et al. (2010). This is done in a multiple regression model with participant age in years, AHI as the independent or predictor variable, and fractional grey matter volume as the dependent or response variable. To correct for the effect of age on fractional grey matter volume we ran a separate regression estimating the effect of age on fractional grey matter using only data from the NSA group. We then estimated an age‐normalized fractional grey matter difference for each participant by subtracting each participant's fractional grey matter volume from the estimated fractional grey matter volume based on their age (using the previous model) (Tsiatis et al. 2008). This value represents the difference between a participant's GMVF and the expected value for a healthy participant given their age. Finally, we ran regressions of the age‐normalized fractional grey matter differences against CBF, SvO_2_, and CMRO_2_ at baseline as well as percent change during breath‐holds. As we are uncertain how these relationships may or may not interact with AHI we used the Akaike information criterion (AIC) as a metric for model selection (Ding et al. 2018). Three models are evaluated for the effect of each neurometabolic parameter on grey matter: one with only the effect of the neurometabolic parameter, one with AHI but no interaction term, and one with an interaction term. Results from the model with the lowest AIC are reported for each neurometabolic parameter.

## 3 Results

### 3.1 Relationship Between AHI and Global Grey Matter Volume Fraction

**FIGURE 2:**
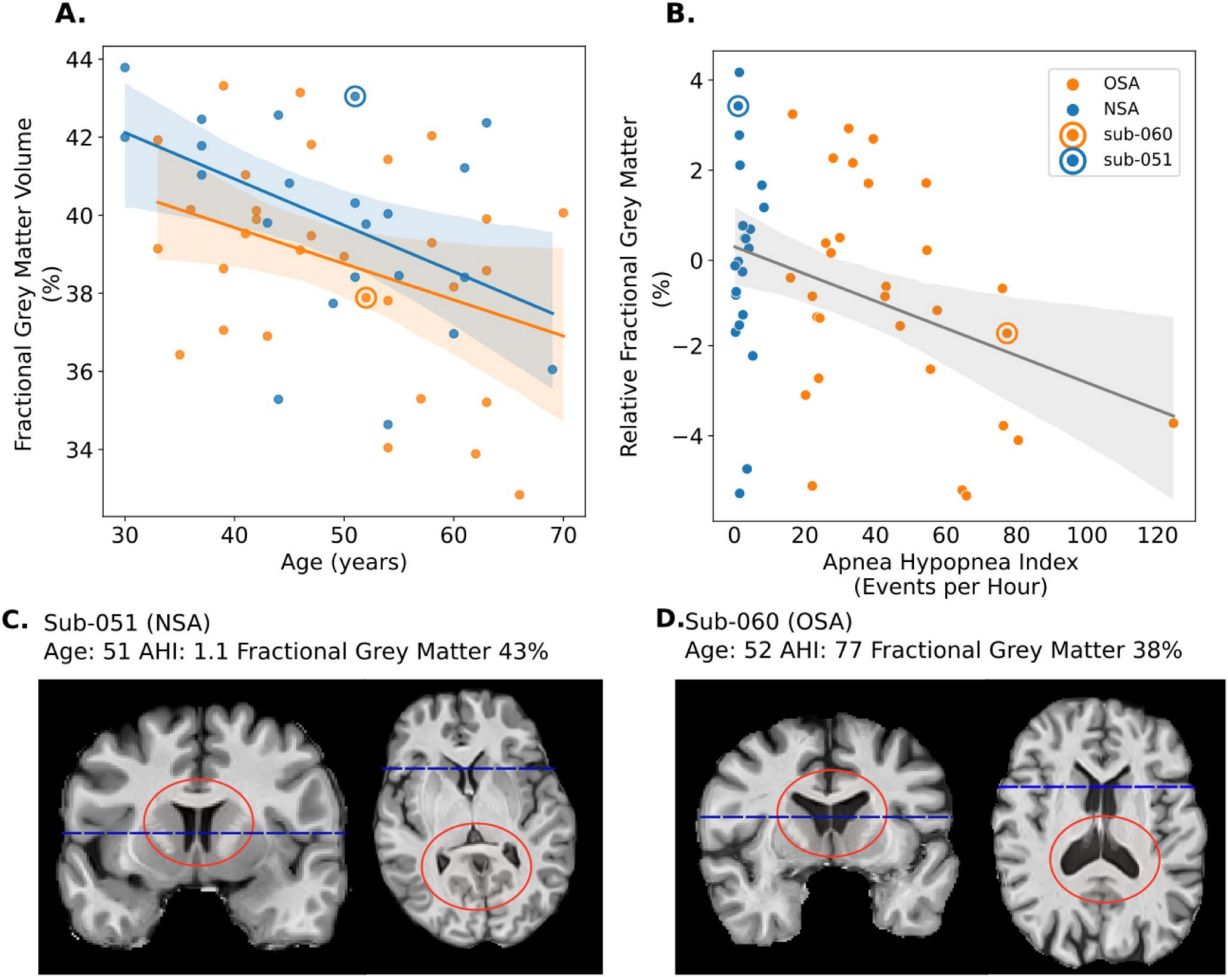
Fractional grey matter volume was associated with age and OSA severity. Lower GMVF is inversely associated with age (displayed in A) in both OSA patients (orange) and NSA controls (blue). Displayed in B is the association between larger values of AHI (x‐axis) with lower values of age‐normalized fractional grey matter (y axis), indicative of atrophy. (C, D) Skull‐stripped T1w MPRAGE structural images displayed for two exemplar participants (circled data points) of the same sex and similar age (coronal and horizontal locations indicated by blue dashed line) c from OSA and NSA groups. Participants' age, AHI, and fractional grey matter are noted above their images. Notice the enlarged ventricles circled (red circles) in the subject of panel D.

Multiple regression to evaluate the effect of age and OSA severity replicated previous results. Age was found to be a significant predictor of fractional grey matter volume, corresponding to a drop in normalized grey matter volume of 0.1% per year of age (see Table 2). The decrease in fractional grey matter volume with age is shown in Figure 2, panel A, separately for both NSA (blue) and OSA (orange) groups. We also find that a significant drop in normalized grey matter was associated with AHI, estimated at −0.03%/(event/h) (shown in Figure 2, panel B after controlling for age). The loss in fractional grey matter volume with age corresponds to a reduction of approximately 1% over the course of a decade while the drop associated with AHI corresponds to a reduction of about 1% for an individual with an AHI of 33. In panels C and D of Figure 2 we display the anatomic images of two male participants of similar age as examples, where enlarged ventricles can be noted for the OSA patient.

**TABLE 2:** Summary of regression results for the associations between age, AHI, and grey matter volume fraction.

Effect	Coefficient (%/unit variable)	SE	95% CI		*p*
			LL	UL	
Age (years)	−0.1004	0.011	−0.161	−0.039	**0.002**
AHI (events/h)	−0.0297	0.030	−0.052	−0.007	**0.011**

*Note:* Negative regression coefficients for age and AHI suggest that lower fractional grey matter volume is associated with greater age and AHI values (*N* = 52). Coefficients (slope) represent the unit change in fractional grey matter volume associated with a unit change in each variable, that is, its dimension is % grey matter/unit variable. Each coefficient is presented along with a standard error of the estimate, a 95% confidence interval, and level of significance via *p*‐value.

Abbreviations: CI, confidence interval; LL, lower limit; SE, standard error; UL, upper limit.

### 3.2 Associations Between Neurometabolic Parameters and Grey Matter Volume Fraction (GMVF)

Analysis of simple correlations between baseline neurometabolic parameters and brain structure showed no significant relationships between age‐normalized GMVF and baseline CBF (*r* = −0.09, *p* = 0.56), SvO_2_ (*r* = −0.17, *p* = 0.27), or CMRO_2_ (*r* = 0.16, *p* = 0.29). Incorporating AHI as an additional variable did not reveal any significant relationships for CBF or SvO_2_ (see Table 3). However, an interaction between AHI and CMRO_2_ on age‐normalized fractional grey matter differences emerged. These results suggest that there is no simple relationship between age‐normalized fractional grey matter and SvO_2_, CBF, and CMRO_2_ at baseline. However, the significant interaction may suggest that for individuals with greater AHI, greater baseline CMRO_2_ is tied to greater fractional grey matter as compared to age‐normed expectations.

**TABLE 3:** Summary of regression analyses for the relationship between age‐normalized grey matter fraction with the three neurometabolic serving as explanatory variables parameters at baseline.

Effect	Coefficient (%/unit variable)	SE	95% CI		*p*
			LL	UL	
Baseline CBF model					
AHI (events/h)	−0.0261	0.013	−0.053	0.001	0.055
CBF (mL/min)/100 g	−0.0113	0.039	−0.090	0.067	0.774
Baseline SvO_2_ model					
AHI (events/h)	0.1567	0.112	−0.070	0.384	0.171
SvO_2_ (%)	0.0390	0.058	−0.078	0.156	0.503
SvO_2_:AHI (interaction)	−0.0027	0.002	−0.006	0.001	0.112
Baseline CMRO_2_ model					
AHI (events/h)	−0.1480	0.055	−0.260	−0.036	**0.011**
CMRO_2_ (μmol/min)/100 g	−0.0235	0.020	−0.064	0.017	0.251
CMRO_2_:AHI (interaction)	0.0014	0.001	0.000	0.003	**0.027**

*Note:* The regression (*N* = 44) coefficients displayed for AHI, each of the neurometabolic parameters, and an interaction term, represent the unit change in age‐normalized fractional grey matter associated with a unit change in the variable on each row. The standard error of the coefficient is also given along with a 95% confidence interval, and *p*‐value to indicate significance. Significant variables and associated statistics are emboldened.

Abbreviations: CI, confidence interval; LL, lower limit; SE, standard error; UL, upper limit.

**FIGURE 3:**
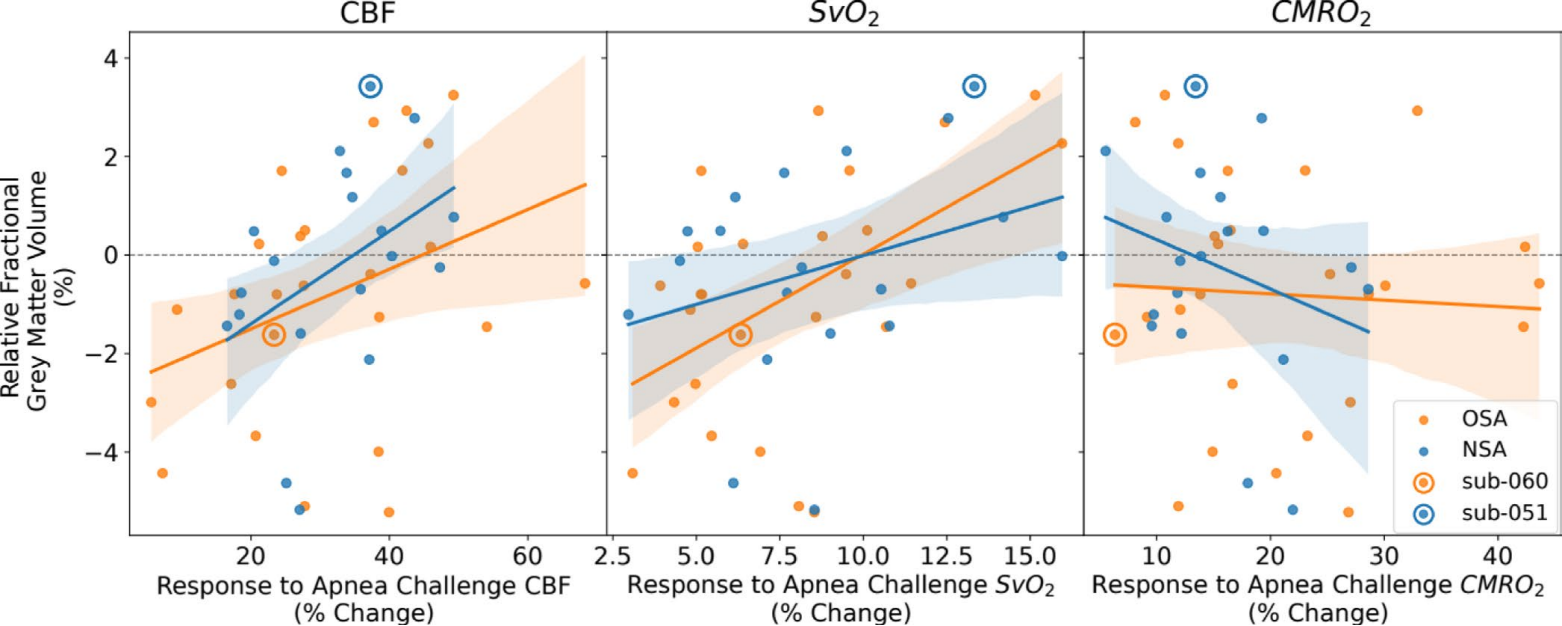
Relationship between age‐normalized grey matter volume fraction and changes in the measured neurometabolic parameters in response to breath‐hold stimuli. Cerebral blood flow (CBF), SvO_2_, and CMRO_2_ are plotted against age‐normalized grey matter volume, that is, the difference between participants' fractional grey matter volume and their expected fractional grey matter volume given their age. The zero line is plotted as a dashed grey line and the data points associated with the example participants displayed in Figure 1 are highlighted with a circle.

Though no significant relationships were identified between baseline neurometabolic parameters and brain grey matter volume fraction, these neurometabolic parameters are known to change under hypercapnic–hypoxic conditions, such as those experienced during breath‐holds. These changes may also mirror the conditions that patients with OSA encounter during sleep. Therefore, we also analysed the relationship between individual grey matter volume changes and individual transient changes in CBF, SvO_2_, and CMRO_2_ during breath‐hold stimuli (shown in Figure 3).

Though the fractional change in CMRO_2_ was significantly associated with AHI (reported in Wu et al. 2022), it did not predict any differences in GMVF. However, significant relationships emerged between age‐normalized fractional grey matter difference and the percent change in SvO_2_ and CBF during breath holds (see Table 4).

**TABLE 4:** Summary of regression analyses testing associations between grey matter volume, AHI, and the transient changes of the three neurometabolic parameters in response to breath‐hold stimulus.

Effect	Coefficient	SE	95% CI		*p*
	(%/unit variable)		LL	UL	
Percent change CBF model					
AHI (events/h)	0.0396	0.038	−0.036	0.115	0.298
CBF (% change)	0.1224	0.042	0.037	0.208	**0.006**
CBF: AHI	−0.0021	0.001	−0.004	−0.001	0.075
Percent change SvO_2_ model					
AHI (events/h)	−0.0189	0.013	−0.044	0.007	0.142
SvO_2_ (% change)	0.2487	0.097	0.053	0.444	**0.014**
% change CMRO_2_ model					
AHI (events/h)	−0.0248	0.013	−0.052	0.002	0.071
CMRO_2_ (% change)	−0.0208	0.037	−0.095	0.053	0.574

*Note:* Regression coefficients represent the unit change in age‐normalized fractional grey matter associated with a unit change in the variable on each row. The standard error of the coefficient is also given along with a 95% confidence interval and *p*‐value to indicate significance. Significant positive coefficients for CBF and SvO_2_ suggest that larger changes in these values during breath‐holds are related to greater relative grey matter fraction. Significant variables and associated statistics are highlighted with bold text.

Abbreviations: CI, confidence interval; LL, lower limit; SE, standard error; UL, upper limit.

## 4 Discussion

This work examines previously collected vascular‐neurometabolic parameters in OSA patients and their relationship to grey matter volume fraction. We found that transient changes in global CBF and SvO_2_ during a breath‐hold task are predictive of grey matter volume changes beyond those predicted by AHI alone. The results provide convergent validity for whole‐brain measures of neurometabolism as an indicator of neural health. While the risk of neurodegenerative disease, including Parkinson's (Sun et al. 2020) and Alzheimer's disease (Bubu et al. 2020), in patients with OSA is already known to increase the morbidity of this highly prevalent disorder (Benjafield et al. 2019), non‐invasive assessment of neurometabolism using a simple breath‐hold paradigm mimicking apneas could complement structural imaging as a further metric of brain health.

Previous research separately examined the association between OSA and increased risk of neurodegenerative disease (Andrade et al. 2018; Sun et al. 2020) and decreased grey matter volume (Joo et al. 2010; Selcuk et al. 2024). In more recent work, the effect of OSA on CMRO_2_, both at baseline and in response to breath‐hold stimuli (Rodgers et al. 2013, 2016) was studied. In the present study, differences in grey matter volume and neurometabolic parameters were evaluated within the same study cohort to ascertain whether neurometabolic parameters can provide information complementary to grey matter loss in patients with OSA. Previously, Wu et al. had shown that the breath‐hold response in CMRO_2_ differed between OSA and NSA participants but not CBF or SvO_2_ as stand‐alones. Here, we found that the response of CBF and SvO_2_ to breath‐hold challenges, mimicking spontaneous apneas, tracked global grey matter volume loss whereas CMRO_2_ did not.

The associations between OSA and brain health have previously been hypothesized to be linked through mechanisms of oxidative stress (Lavie 2015), in turn causing neurometabolic dysfunction (Lushchak et al. 2021). Not only do individuals with OSA have lower levels of CMRO_2_ at baseline, but the relationships we find between changes in neurometabolic parameters in response to volitional apneas and grey matter loss provide convergent validity for these measures as a quantitative indicator of neurological health. Specifically, we find that changes to SvO_2_ and CBF in response to a breath‐hold stimulus are related to age‐corrected measures of grey matter volume fraction in both OSA patients and NSA controls. While our cross‐sectional design and absence of clinical outcome measures preclude conclusions about whether neurometabolic dysfunction mediates the relationship between OSA and neurodegenerative disease, the results provide a rationale and possible tools to study this relationship. The relationship we found between changes in neurometabolic parameters in CBF, SvO_2_ and grey matter deficits suggests the potential of MRI‐based brain oximetry to provide an additional non‐invasive quantitative measure of neurological health.

However, we note some limitations. The first is that the cross‐sectional and observational nature of the data precludes claims about the causality of the observed metabolic responses, OSA severity, and grey matter volume fraction. Future studies incorporating neurocognitive outcomes and longitudinal observations may provide further insights into the clinical relevance of our observations. While disruptions to neurometabolism have been proposed as a plausible mechanism to mediate the relationship between OSA and neurodegenerative disease, we can only draw inferences about correlation, not causation, as other confounding variables may exist. Specifically, we are unable to address whether OSA has a cumulative impact, which could be confounded with AHI as there is progression of adverse effects with disease duration (Pendlebury et al. 1997). However, determining individual onset, and therefore duration, can be challenging as OSA often goes undiagnosed (Simpson et al. 2013). Second, spontaneous apneas occur during sleep only. Nevertheless, the breath‐hold paradigm performed in awake participants causes a hypercapnic‐hypoxic response analogous to that observed in spontaneous apneas during sleep (i.e., arterial desaturation, increased SvO_2_ and elevated CBF) (Wu et al. 2022). Still, there are other features of natural apneas that are not replicated with this paradigm. Specifically, sleep results in neurovascular‐metabolic uncoupling (Schneider et al. 2019; Dennison et al. 2025), which may further modulate the responses to transient hypoxia from apneas during sleep. Third, while we consider the relationship between GMVF and the neurometabolic response to breath‐hold stimuli an indicator of general neurological health, it is plausible that the breath‐hold response is driven disproportionately by grey matter. If this is the case, the neurometabolic information may provide little additional information beyond what can be gleaned from grey matter volume alone.

Nevertheless, our findings suggest that neurometabolic responses to breath‐hold stimuli may provide additional insight into neural health beyond traditional measures of OSA severity (i.e., AHI and arterial desaturation). Therefore, longitudinal evaluation would be needed to determine whether these alterations contribute as a causal mechanism in disease progression, or what potential clinical utility the method might hold. Additionally, even though the transient suspended respiration paradigm in the form of cued breath‐holds provides some of the hypoxic conditions experienced by OSA patients during sleep, volitional apneas may not fully replicate the conditions of sleep apnea. Given that sleep alters the coupling between CBF and oxygen extraction, the transient apneic changes of CBF and arterial and venous saturation levels experienced by patients are superimposed onto the sleep‐dependent changes, that is, the sleep‐stage dependent decrease in CMRO_2_ (Xu et al. 2024). Therefore, while very recent preliminary work by the present authors which monitors the neurometabolic changes in OSA patients as they experience spontaneous apneas during sleep has been reported (Dennison et al. 2025), the dynamics of these events will eventually have to be tied to structural changes in brain architecture.

In conclusion, our data suggest that neurometabolic responses to a cued breath‐hold task, that is, transient changes in CBF and SvO_2_, are associated with global grey matter volume fraction in individuals with OSA. Additionally, the relationship between OSA severity, as expressed by AHI and age‐normalized fractional grey matter difference, is no longer significant when including information about changes in CBF and SvO_2_ during breath holds, suggesting a mediating role for neurometabolic dysfunction in the relationship between OSA and neurodegeneration. However, longitudinal studies are required to establish whether the abnormal breath‐hold response we observe precedes, or follows, neurodegenerative changes within a patient. Lastly, we find that the neurometabolic breath‐hold response is predictive of grey matter in NSA participants as well, providing convergent validity for temporally resolved brain oximetry, yielding metrics of neurometabolism as non‐invasive quantitative indicators of neuronal health. Expanding these investigations to larger and more diverse cohorts will be critical to understanding whether parameters of neurometabolic dysregulation represent a modifiable target for reducing neurodegenerative risk in OSA populations.

## 5 Conclusions

Obstructive sleep apnea (OSA) is a common condition linked to an increased risk of neurodegeneration, yet the pathways mediating this relationship are unclear. Prior studies have shown OSA to be associated with altered brain metabolism and reduced GMVF. The present results provide an assessment of both factors within the same cohort, revealing that neurometabolic responses to apnea‐like stimuli can track deficits in GMVF in both OSA patients and healthy controls, highlighting a potential mechanism underlying the brain's vulnerability to OSA. Understanding this link could inform future research and help to identify individuals at risk for clinical neurodegeneration earlier.

## Author Contributions

**Jeffrey B. Dennison:** writing – original draft, formal analysis, data curation, conceptualization, methodology, visualization, software. **Pei‐Hsin Wu:** formal analysis. **Michael C. Langham:** methodology, supervision, visualization, writing – review and editing. **Richard J. Schwab:** writing – review and editing. **John A. Detre:** writing – review and editing. **Felix W. Wehrli:** resources, funding acquisition, writing – review and editing, conceptualization, supervision, data curation.

## Ethics Statement

Ethical approval for this study was obtained from the Institutional Review Board (IRB) at the University of Pennsylvania (IRB # 820858).

## Consent

Informed consent was obtained from all volunteers in the study including patients.

## Conflicts of Interest

Dr. Schwab reports grant and/or research support from ResMed, Inspire, and CryOSA, royalties from UpToDate and Merck Manual, research consulting for Eli Lilly, and Medical Advisory Board membership for eXciteOSA, Onera and Sleep Evolution. The remaining authors declare no conflicts of interest.

## Supporting information


**Data S1:** Supporting Information.

## Data Availability

Data available upon request from the authors.
