# Supplementary material for: Neurometabolic Response to Apneic Stimuli Tracks Global Grey Matter Volume Deficits in Patients With Obstructive Sleep Apnea
Source: J Sleep Res. 2025 Nov 19;35(3):e70250. doi: 10.1111/jsr.70250 (PMC13193465; doi:10.1111/jsr.70250)
Supplement: Supplementary file 1 — Data S1: Supporting Information. [file JSR-35-e70250-s001.docx]

*S.1. MRI Pulse Sequence and Experiment Protocol*

The specific pulse sequence (termed OxFlow) and experimental procedure are described in-depth in previous reports [1, 2]. Briefly, imaging was performed at the University of Pennsylvania using a 3 Tesla MRI system (Prisma, Siemens Healthineers, Erlangen, Germany) with a product 20-channel head/neck coil. A T1-weighted magnetization-prepared rapid gradient-echo (MP-RAGE) was taken for brain volume quantification (1 mm isotropic resolution). MRI quantification of neurometabolism was achieved via interleaving equal and opposite velocity-encoded multi-echo gradient-echoes at the level of the superior sagittal sinus (SSS) as described previously [1]. Measuring at the level of the SSS allows for the estimation of average venous blood oxygenation for the entire brain and provides a large vessel with a stable relationship to total blood flow for the brain [3]. Time-of-flight MR angiograms had been collected for slice prescription of the sequence at the SSS and computation of the tilt angle θ necessary for calculating SvO_2_. (see [4]). Velocity and field maps were produced every 1.29 seconds for a total of 9.5 minutes.

**References**

1. Cao, W., et al., *High-speed whole-brain oximetry by golden-angle radial MRI.* Magn Reson Med, 2018. **79**(1): p. 217-223.
2. Wu, P.H., et al., *MRI evaluation of cerebrovascular reactivity in obstructive sleep apnea.* J Cereb Blood Flow Metab, 2020. **40**(6): p. 1328-1337.
3. Caporale, A.S., et al., *Superior sagittal sinus flow as a proxy for tracking global cerebral blood flow dynamics during wakefulness and sleep.* J Cerebral Blood Flow Metab, 2023.
4. Wehrli, F.W., et al., *Time-resolved MRI oximetry for quantifying CMRO(2) and vascular reactivity.* Acad Radiol, 2014. **21**(2): p. 207-14.
